# Itaconate alleviates anesthesia/surgery-induced cognitive impairment by activating a Nrf2-dependent anti-neuroinflammation and neurogenesis via gut-brain axis

**DOI:** 10.1186/s12974-024-03103-w

**Published:** 2024-04-22

**Authors:** Xiangyi Kong, Wenyuan Lyu, Xiaojie Lin, Chunlong Lin, Hao Feng, Lin Xu, Kaiyue Shan, Penghui Wei, Jianjun Li

**Affiliations:** 1 Department of Anesthesiology, Qilu Hospital (Qingdao), Cheeloo College of Medicine, Shandong University, 758 Hefei Road, Qingdao, China; 2 Laboratory of Anesthesia and Brain Function, Qilu Hospital (Qingdao), Cheeloo College of Medicine, Shandong University, 758 Hefei Road, Qingdao, China

**Keywords:** Itaconate, 4-octyl itaconate, Postoperative cognitive dysfunction, Neuroinflammation, Neurogenesis, Gut microbiota, Nrf2

## Abstract

**Background:**

Postoperative cognitive dysfunction (POCD) is a common neurological complication of anesthesia and surgery in aging individuals. Neuroinflammation has been identified as a hallmark of POCD. However, safe and effective treatments of POCD are still lacking. Itaconate is an immunoregulatory metabolite derived from the tricarboxylic acid cycle that exerts anti-inflammatory effects by activating the nuclear factor erythroid 2-related factor 2 (Nrf2) pathway. In this study, we investigated the effects and underlying mechanism of 4-octyl itaconate (OI), a cell-permeable itaconate derivative, on POCD in aged mice.

**Methods:**

A POCD animal model was established by performing aseptic laparotomy in 18-month-old male C57BL/6 mice under isoflurane anesthesia while maintaining spontaneous ventilation. OI was intraperitoneally injected into the mice after surgery. Primary microglia and neurons were isolated and treated to lipopolysaccharide (LPS), isoflurane, and OI. Cognitive function, neuroinflammatory responses, as well as levels of gut microbiota and their metabolites were evaluated. To determine the mechanisms underlying the therapeutic effects of OI in POCD, ML385, an antagonist of Nrf2, was administered intraperitoneally. Cognitive function, neuroinflammatory responses, endogenous neurogenesis, neuronal apoptosis, and Nrf2/extracellular signal-related kinases (ERK) signaling pathway were evaluated.

**Results:**

Our findings revealed that OI treatment significantly alleviated anesthesia/surgery-induced cognitive impairment, concomitant with reduced levels of the neuroinflammatory cytokines IL-1β and IL-6, as well as suppressed activation of microglia and astrocytes in the hippocampus. Similarly, OI treatment inhibited the expression of IL-1β and IL-6 in LPS and isoflurane-induced primary microglia in vitro. Intraperitoneal administration of OI led to alterations in the gut microbiota and promoted the production of microbiota-derived metabolites associated with neurogenesis. We further confirmed that OI promoted endogenous neurogenesis and inhibited neuronal apoptosis in the hippocampal dentate gyrus of aged mice. Mechanistically, we observed a decrease in Nrf2 expression in hippocampal neurons both in vitro and in vivo, which was reversed by OI treatment. We found that Nrf2 was required for OI treatment to inhibit neuroinflammation in POCD. The enhanced POCD recovery and promotion of neurogenesis triggered by OI exposure were, at least partially, mediated by the activation of the Nrf2/ERK signaling pathway.

**Conclusions:**

Our findings demonstrate that OI can attenuate anesthesia/surgery-induced cognitive impairment by stabilizing the gut microbiota and activating Nrf2 signaling to restrict neuroinflammation and promote neurogenesis. Boosting endogenous itaconate or supplementation with exogenous itaconate derivatives may represent novel strategies for the treatment of POCD.

## Introduction

Postoperative cognitive dysfunction (POCD) is defined as long-term cognitive impairment that occurs weeks to months after surgery. The incidence of POCD in major non-cardiac surgery patients aged > 65 years has been reported to be 25.8% at one week and 9.9% at three months postoperatively [1–4]. Although the exact pathophysiology behind the development of POCD is not fully understood, it is believed to be caused by the following mechanisms: blood–brain barrier damage, neuroinflammatory responses, abnormal synaptic transmission, neuronal apoptosis, oxidative stress, abnormal amyloid-beta deposition and Tau protein phosphorylation, polymorphisms of the apolipoprotein E gene, and abnormal energy metabolism [5–7]. In addition, long-standing geriatric POCD can be a consequence of Alzheimer’s disease, decreasing quality of life and imposing a significant economic burden on families and society [8]. With the growing elderly population worldwide, the need for surgical procedures is rapidly increasing, leading to a high prevalence of POCD. However, our understanding and diagnosis of POCD still encounter numerous challenges. Therefore, there is an urgent need to explore the pathogenesis of POCD from a new perspective and identify new targets for drug therapy.

Itaconate is an endogenous metabolite derived from the tricarboxylic acid cycle and is produced by cis-aconitic acid decarboxylation catalyzed by immune response gene 1 (IRG1) in the mitochondrial matrix [9]. Itaconate is an immunoregulatory metabolite that activates the nuclear factor erythroid 2-related factor 2 (Nrf2) pathway. In recent years, it has been shown that itaconate can attenuate neuroinflammation and exert dopamine neuroprotection in Parkinson’s disease through inhibition of NLR-family pyrin domain-containing protein 3 (NLRP3) inflammasome. The mechanism of this neuroprotective effect of itaconate involves the scaffold protein p62/Nrf2/Heme oxygenase 1 (HO-1)/Nuclear factor kappa-light-chain-enhancer of activated B cells (NF-κB) axis pathway in microglia [10, 11]. Itaconate regulates metabolic remodeling and inflammation in macrophages by inhibiting succinate dehydrogenase [12]. High intracellular concentrations of endogenous itaconate accumulate in activated macrophages [13]. However, itaconate has a low secretion rate. Despite the occurrence of acute inflammation and sepsis, high concentrations of itaconate have not been detected in humans [14]. Therefore, exogenous itaconate might be an effective treatment to exert therapeutic effects. It has been shown that the infusion of itaconate exerts neuroprotective effects against cerebral ischemia/reperfusion and traumatic brain injury [15]. However, the effects of itaconate on POCD remain unclear. 4-octyl itaconate (OI), a cell and blood–brain barrier-permeable itaconate derivative, exhibits similar thiol reactivity to itaconate and has been shown to be a suitable endogenous itaconate surrogate [9, 16]. In this study, we explored the role of OI in ameliorating POCD in both in vivo and in vitro models, as well as the underlying molecular mechanisms.

## Materials and methods

### Animals

All animal experiments were approved by the Animal Committee of Shandong University Qilu Hospital (Qingdao, China) (KYDWLL-202107). 18-month-old C57BL/6 male mice were used in the study and housed in a 23–25 °C, 12-h light–dark cycle environment, and all mice were allowed to eat and drink freely (2–3 mice/cage). A total of 162 aged mice were utilized in the study. Mice were randomly assigned to Intervention groups. The number of mice that contributed data for analysis in each experiment was specified in the figure legends.

### Anesthesia and surgery

**Fig. 1:**
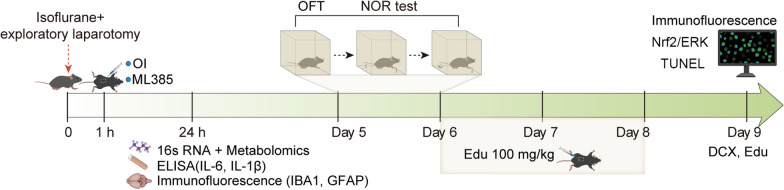
Experimental protocols. Aged mice were administered intraperitoneally with 4-Octyl itaconate (OI) or/and ML385 (an antagonist of Nrf2) 1 h after exploratory laparotomy under isoflurane anesthesia. Intestinal contents, blood, and brain tissue were collected for 16S rRNA gene sequencing, metabolomics, ELISA, and immunofluorescence analysis 24 h after surgery. Another cohort of aged mice underwent the open-field test (OFT) and new object recognition (NOR) test on postoperative days 5 and 6. Subsequently, 5-ethynyl-2′-deoxyuridine (EDU) was intraperitoneally injected into aged mice for 3 days, and brain tissue was collected for immunofluorescence analysis 9 days after surgery

A modified aseptic exploratory laparotomy was performed under isoflurane anesthesia in a chamber prefilled with 3.5% isoflurane and 100% oxygen while maintaining spontaneous ventilation. After the mice lost their upright reflex, they were placed on their right side and anesthetized using 1.5% isoflurane for 20 min. Surgery was performed under 1.5–2% isoflurane anesthesia, and an oxygen mask was worn during surgery. A 1-cm midline vertical incision was made in the abdomen to penetrate and explore the abdominal cavity. The operator manipulated the organs and organ musculature and removed approximately 2 cm of the intestine, which was rubbed vigorously with two sterile swabs for 30 s. The bowel was placed back into the abdominal cavity and the wound was closed with a 4–0 sterile surgical suture. Polysporin (Johnson & Johnson Inc.) was used to relieve and prevent postoperative pain. The duration of surgery was 15 min, the total duration of isoflurane anesthesia was 1.5 h. The sham-operated mice were anesthetized with isoflurane, and the abdominal area was shaved and cleaned as described above, without making any incision. OI (50 mg/kg, Cat# SML2338, Sigma-Aldrich) was administered intraperitoneally immediately 1 h after surgery. The dosage of OI used in the study were based on previous reports [17–19] and our pilot study. In addition, for the Nrf2 antagonist treatment, aged mice received intraperitoneal injections of ML385 (30 mg/kg, Cat# SML1833, Sigma-Aldrich), a specific Nrf2 antagonist, 1 h after surgery [20], and the same amount of dimethyl sulfoxide and normal saline were administered intraperitoneally to mice in other groups. 5-ethynyl-2′-deoxyuridine (EDU) (100 mg/kg, C0081L, Beyotime) was intraperitoneally administered for three consecutive days before the euthanasia of a separate cohort of aged mice. A schematic illustration of the experimental procedure is outlined in Fig. 1.

### Cell culture and treatment

Fetal mice were removed at 17 days of gestation, and the heads were cut off and placed in ice-cold Hank’s balanced salt solution (HBSS). The brain tissues were separated with micro tweezers and placed into another Petri dish containing ice-cold HBSS. The meninges were peeled off with microforceps, and the hippocampal tissue was separated and transferred to a centrifuge tube containing serum-free Dulbecco’s modified Eagle medium (DMEM). The tubes were centrifuged at 1000 rpm for 5 min, the supernatant was removed by aspiration, and 1 mL of 0.05% tryptic digest was added to each centrifuge tube, which was lightly inverted several times and then digested for 20 min in a 37 °C incubator. The reaction was terminated by adding serum-containing medium to each tube, and the tubes were inverted lightly until the brain tissue was separated. After a 5-min centrifugation at 1000 rpm, the supernatants were discarded, and the pellet was resuspended with medium. Glass coverslips were coated with poly-D-lysine for 6 h and then washed with sterile phosphate-buffered saline (PBS) three times. The cells were applied to the cover slips, and the medium was half-exchanged the following day. The cells were cultured for 14 days for experiments.

### Incubation with lipopolysaccharide (LPS) and isoflurane

Primary microglia and hippocampal neurons were placed in an incubator at 37 °C. Cells were treated with 1 μg/mL of LPS and exposed to 3% isoflurane in pure oxygen for 6 h. The isoflurane concentration was continuously monitored during this period using a Datex-Ohmeda ULT-SV analyzer. Primary microglia and neurons were treated with 250 μM OI or 5 μM ML385 1 h after POCD modeling, and the cells were collected after 24 h for experimental assays [21].

### Enzyme-linked immunosorbent assay (ELISA)

The cytokines IL-6 and IL-1β secreted by microglial culture supernatant and from the hippocampus of aged mice were assessed 24 h post-operation using ELISA kits, according to the manufacturer’s instructions (Elabscience) [22, 23].

### Ultra-high performance liquid chromatography-mass spectrometry/mass spectrometry analysis

Twenty-four hours after surgery, the intestinal contents of Sham, POCD, and POCD + OI groups were collected and weighed. The samples were placed in a 4 °C autosampler throughout the analysis. The samples were analyzed using a SHIMADZU-LC30 ultra-high performance liquid chromatography (UHPLC) with an ACQUITY UPLC®HSS T3 (2.1 × 100 mm, 1.8 µm) column (Waters, Milford, MA, USA). The injection volume was 4 μL, the column temperature was 40℃, and the flow rate was 0.3 mL/min. The chromatographic mobile phase A was a 0.1% formic acid solution, and mobile phase B was an acetonitrile solution. The chromatographic gradient elution program was as follows: 0–2 min, 0 B; 2–6 min, B varied linearly from 0 to 48%; 6–10 min, B varied linearly from 48 to 100%; 10–12 min, B was maintained at 100%; 12–12.1 min, B varied linearly from 100 to 0%; 12.1–15 min, B was maintained at 0%.

The positive (+) and negative (−) modes of each sample were detected by electrospray ionization. The samples were separated by UPLC, and mass spectrometry analysis was performed using a QE Plus mass spectrometer (Thermo Scientific). Ionization was performed using a heated electrospray ionization source. Ionization conditions were: spray voltage, 3.8 kv ( +) and 3.2 kv ( −); capillary temperature, 320 ± ; sheath gas, 30 ( ±); auxiliary gas, 5 ( ±); probe heater temperature, 350 ( ±); S-Lens RF level 50; mass spectrometry acquisition time, 15 min; parent ion scanning range: 75–1050 m/z; primary mass spectral resolution, 70,000@m/z 200; AGC target, 3e6; and primary maximum IT, 100 ms. Secondary mass spectrometry analysis was performed according to the following conditions: secondary mass spectra (MS2 scans) of the 10 highest intensity parent ions were triggered after each full scan, secondary mass spectral resolution: 17,500@m/z 200, AGC target: 1e5, 2-stage maximum IT: 50 ms, MS2 activation type: HCD, isolation window: 2 m/z, normalized collision energies (step): 20, 30, and 40.

### Microbial taxonomy and metabolite biomarker correlation analysis

Microbiome bioinformatic analysis was performed using QIIME 2 2019.4, according to the official tutorial (https://docs.qiime2.org/2019.4/tutorials/). Briefly, the raw sequence data were demultiplexed using the Demux plugin, followed by primer cutting using the CutAdapt plugin. The sequences were then quality-filtered, denoised, merged, and the chimera was removed using the DADA2 plugin. Non-singleton amplicon sequence variants (asv) were aligned with MATT and used to construct phylogenies using fasttre2. α-Diversity metrics (Chao1, Observed species, Shannon, Simpson, Faith’s phylogenetic diversity, Pielou’s uniformity, and Good’s coverage) and β-diversity metrics (weighted UniFrac, unweighted UniFrac, Jaccard distance and Bry–Curtis dissimilarity) were estimated using diversityplugin. The Greengenes 13_8 99% operational taxonomic unit reference sequence was classified using the classiy-sklearn naïve Bayesian classifier in the feature classifier plugin.

### Terminal deoxynucleotidyl transferase dUTP nick end labeling (TUNEL) assay and immunofluorescence staining

Frozen sections were fixed with 4% paraformaldehyde (PFA) for 30–60 min, washed twice with PBS for 10 min each, incubated with 0.5% Triton X-100 at room temperature for 5 min, mixed with terminal deoxynucleotidyl transferase, fluorescent labeling solution, and TUNEL detection solution according to the manufacturer’s instructions (C1088, Beyotime), incubated at room temperature for 60 min, washed three times with PBS, and blocked for confocal microscopy. The slices were sealed and observed under a confocal microscope.

### Immunofluorescence

Brain tissues were fixed with 4% PFA and sectioned in 40-μm cryosections, rinsed 3 times in PBS, blocked in bovine serum album (BSA) for 1 h, and incubated with primary antibodies against Nrf2 (rabbit, 1:500, ab31163, Abcam), Iba1 (rabbit, 1:500, ab178846, Abcam), GFAP (rabbit, 1:500, ab7260, Abcam), EDU (C0081L, Beyotime), doublecortin (DCX) (mouse, 1:100, Santa Cruz Biotechnology, sc-271390), extracellular signal-related kinases (ERK) (phosphoT202/T185, rabbit, 1:300, ab201015, Abcam), MAP2 (mouse, 1:1000, ab300645, Abcam) overnight at 4 °C. The tissues were washed 3 times in PBS, incubated with goat anti-mouse Alexa Fluor^®^ 647 (1:500, ab150119, Abcam),, goat anti-rabbit Alexa Fluor^®^ 488 (1:500, ab150081, Abcam), goat anti-mouse Alexa Fluor^®^ 488 (1:500, ab150117, Abcam) or goat anti-rabbit Alexa Fluor^®^ 647 (1:500, ab150083, Abcam) for 2 h, washed 3 times with PBS, sealed, and dried at 30–32 °C for 10–15 min for confocal imaging (Thermo Fisher, USA). Cellular immunofluorescence was performed as follows: immersed in 4% PFA for 15 min, blocked with BSA for 2 h, incubated with primary antibodies against Nrf2 (rabbit, 1:100, ab31163, Abcam), ERK (phosphoT202/T185, rabbit, 1:1000, ab201015, Abcam) and MAP2 (rabbit, 1:800, ab300645, Abcam) overnight, washed three times with PBS, and incubated with secondary antibody (AlexaFluor488- or AlexaFluor647-goat anti-rabbit IgG) for 2 h. The cells were then rinsed thrice with PBS. The cover glass was used for a Leica confocal microscope (Leica Camera, STELLARIS 5). The same settings were used for image processing [24].

### Open-field test (OFT) and new object recognition (NOR) test

Behavioral testing was performed in an opaque square chamber on a white table above the ground (400 L × 400 mm W × 400 mm H) under dim light and quiet conditions. The NOR test was performed after the OFT. All mice underwent a 10-min acclimatization period in the apparatus on the first day, during which open-field data was recorded. The following day, two identical objects were placed in the apparatus to eliminate any potential presence of unique odors. The mice were positioned approximately 10 cm away from the edge of the apparatus, facing the center at an equal distance. A camera was set up to capture and record the exploration time of the two objects, defined as when their noses were within 2 cm of the objects or when they made direct contact with the objects. The number of times and length of time the mice explored the two objects were recorded for 5 min. To evaluate the effect on memory improvement, a 1-h interval passed after the completion of 5 min of recording. After the dwell time was completed, one of the objects was replaced by another new object in the apparatus, and the experiment was repeated for 5 min using the above experimental method. The exploration time of the mice was observed and recorded (“N” for the novel object and “O” for the old object). The NOR test was quantified as a discrimination index (N/N + O).

### Statistical analysis

The sample sizes for each experiment were determined based on previous studies utilizing similar experimental paradigms. Large sample sizes were employed for behavioral experiments [25, 26], while small sample sizes were utilized for biochemical studies [9]. Statistical analyses were performed using GraphPad Prism software (version 8.0). All data were expressed as mean ± SD. A t-test was used to compare the statistical significance of two independent groups. One-way analysis of variance (ANOVA) followed by Tukey’s test was used to determine differences between groups. For the unpaired distribution and ANOVA, a rank-sum test was performed to check for significance. *P* < 0.05 was considered statistically significant [27].

## Results

### OI treatment alleviates cognitive impairment induced by anesthesia and surgery

**Fig. 2:**
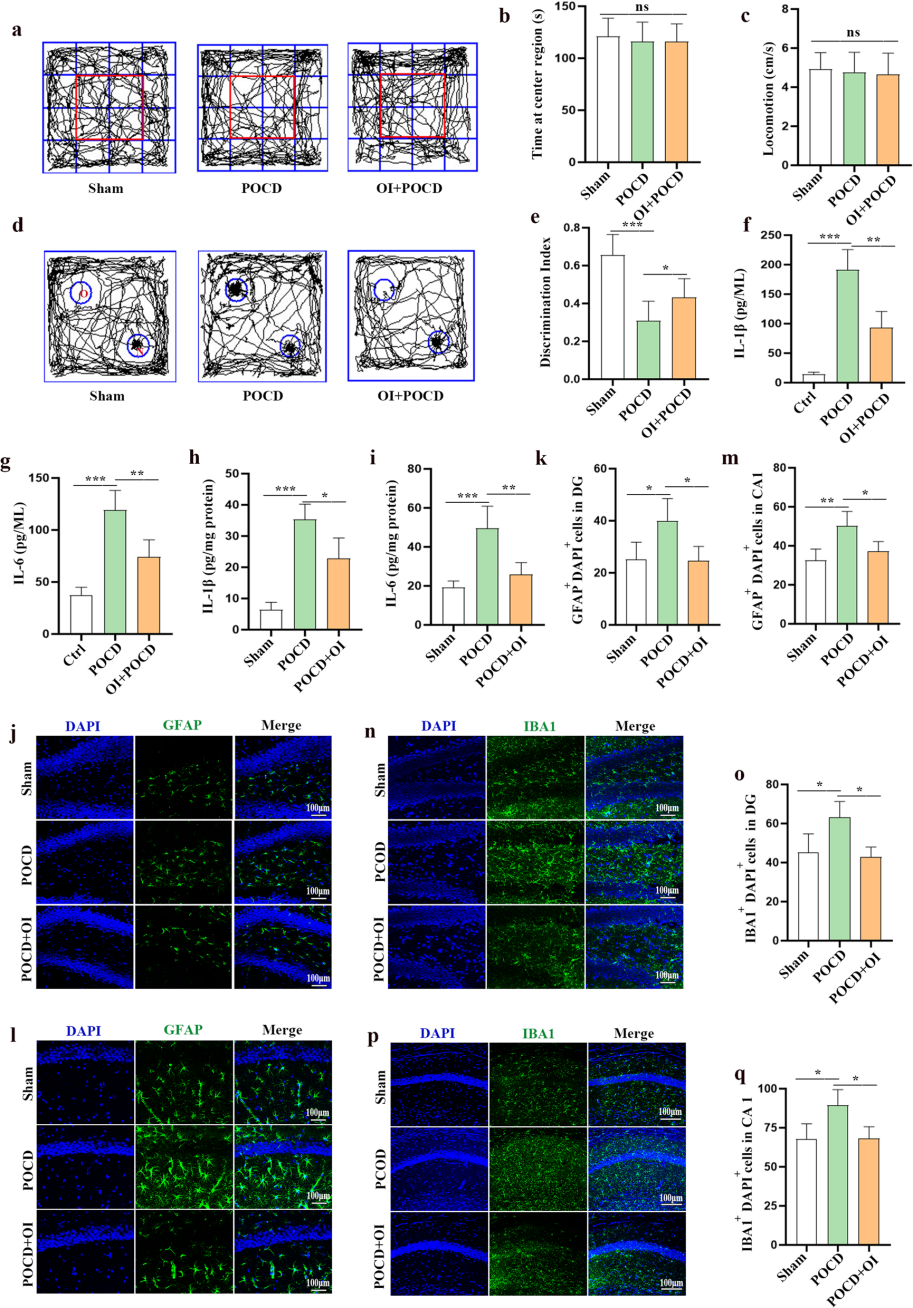
OI treatment alleviates POCD and inhibits hippocampal neuroinflammation in aged mice. a-c Open field test (OFT) was used to detect anxiety and motor activity in aged mice; **a** Road map of the OFT, **b** Time spent in the center region, **c** average velocity in the OFT. **d**, **e** The NOR test was performed to measure the recognition memory; **d** The typical routes in the NOR tests, **e** Discrimination index. Determination of IL-1β (**f**) and IL-6 (**g**) levels by ELISA in primary microglia. Determination of IL-1β (**h**) and IL-6 (**i**) levels by ELISA in the hippocampus of aged mice. Representative immunofluorescence staining and statistics of GFAP (**j**–**m**) and Iba1 (**n**–**q**) in the hippocampal DG and CA1 region of aged mice. Data are expressed as mean ± SD, *n* = 12 for behavioral experiments, *n* = 4 for inflammatory cytokines and immunofluorescence staining analysis, *P < 0.05, **P < 0.01, and ***P < 0.001 by one-way ANOVA. ns, no significant difference

Exploratory laparotomy was performed in mice under isoflurane anesthesia. OI was injected intraperitoneally 1 h after the exploratory laparotomy, and the remaining groups of mice were injected with an equal volume of saline. Behavioral tests were performed in mice using the OFT and NOR tests on postoperative days 5 and 6. No significant difference was observed in the time spent in the center region (F _(2, 33)_ = 0.3357, *P* = 0.7172) and locomotion (F _(2, 33)_ = 0.2165, *P* = 0.8065) among the three groups during the OFT, suggesting that exploratory laparotomy under isoflurane anesthesia and OI treatment did not significantly increase anxiety behaviors or impair motor ability in aged mice (Fig. 2a–c). The data from the NOR test indicated cognitive function in aged mice was impaired after anesthesia and surgery (0.6558 ± 0.1087 vs 0.3083 ± 0.1033, *P* < 0.0001) and cognitive function improved after OI treatment (0.3083 ± 0.1033 vs 0.4325 ± 0.09827, *P* = 0.0160) (Fig. 2d, e). To demonstrate whether OI treatment plays an important role in attenuating neuroinflammation, a hallmark of POCD, we examined the levels of IL-1β and IL-6 in primary hippocampal microglia in vitro 24 h after LPS and isoflurane treatment (Fig. 2f, g) and in hippocampal tissues in vivo 24 h after surgery (Fig. 2h, i). The results showed that OI significantly decreased levels of L-1β (93.37 ± 27.35 vs 191.1 ± 34.37 pg/ml, *P* = 0.0011) and IL-6 (74.16 ± 16.35 vs 119.1 ± 19.06 pg/ml, *P* = 0.0061) in primary microglia subjected to LPS and isoflurane treatment. Similarly, expression of IL-1β (22.83 ± 6.550 vs 35.37 ± 4.870 pg/mg, *P* = 0.0142) and IL-6 (25.75 ± 6.203 vs 49.50 ± 11.36 pg/mg, *P* = 0.0047) in the hippocampus was reduced after OI treatment. Immunofluorescence analysis revealed that the activation of microglia and astrocytes identified by Iba1 (DG, 42.83 ± 5.139 vs 62.99 ± 8.336 cells, *P* = 0.0147; CA1, 68.12 ± 7.588 vs 89.32 ± 10.10 cells, *P* = 0.0259) and GFAP (DG, 24.67 ± 5.437 vs 40.00 ± 8.535 cells, *P* = 0.0317; CA1, 37.26 ± 4.909 vs 50.23 ± 7.344 cells, *P* = 0.0364) labeling, respectively, were significantly reduced in the POCD + OI group compared to the POCD group (Fig. 2j–k). Therefore, OI treatment improved anesthesia/surgery-mediated cognitive impairment and inhibited the postoperative neuroinflammatory responses.

### OI ameliorates intestinal dysbiosis in aged mice with POCD

**Fig. 3:**
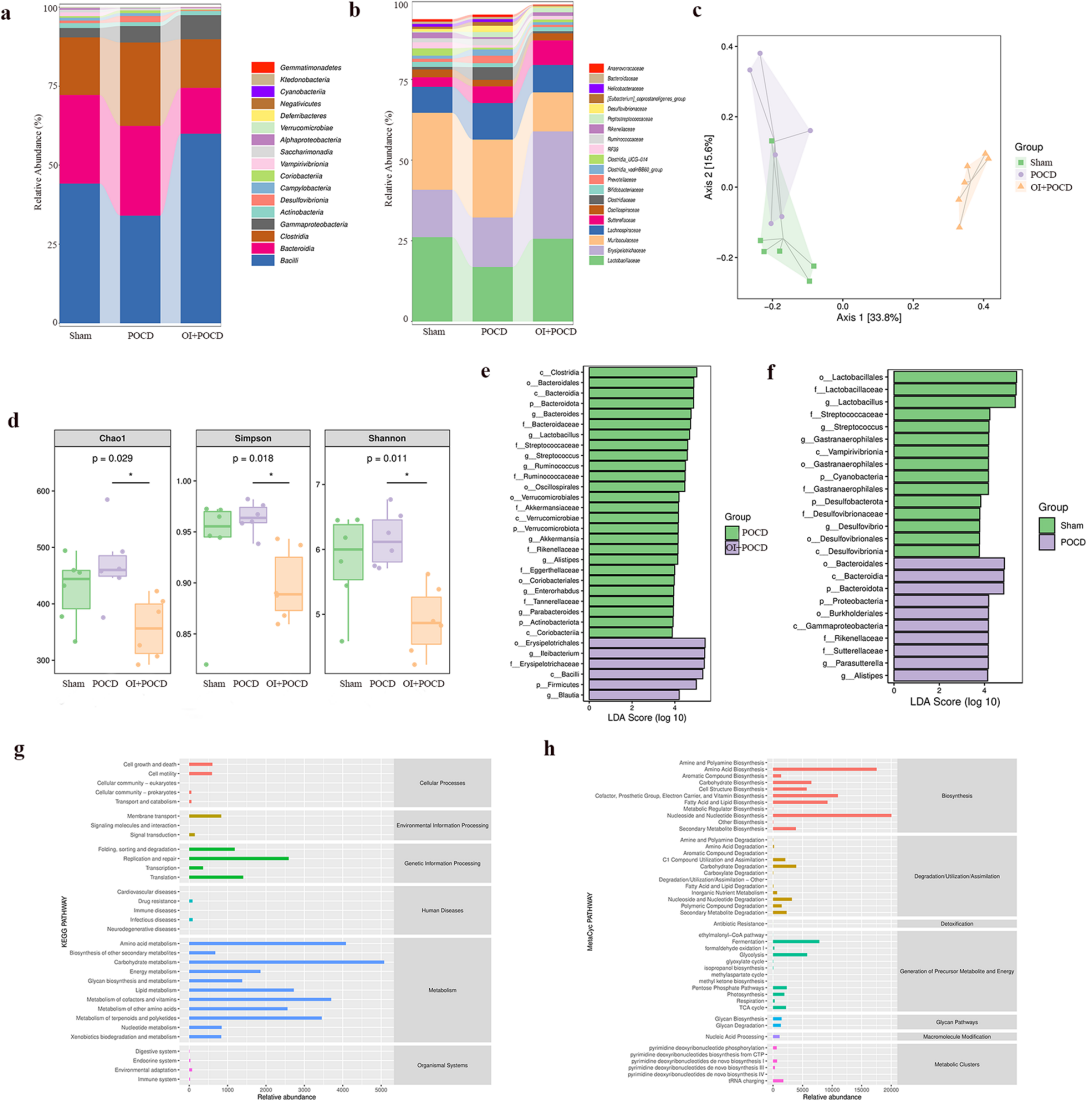
OI improves intestinal dysbiosis in aged mice after anesthesia and surgery. The intestinal contents of aged mice were subjected to 16S rRNA gene sequencing. **a**, **b** Differences in the intestinal flora composition of aged mice among the three groups of aged mice; **a** Relative abundance in the Phylum levels, Negativicutes and Bacteroidia increased after POCD and decreased after OI treatment, while Bacilli decreased after POCD and increased after OI treatment. **b** Relative abundance in the Genus levels, Lactobacillaceae and Erysipelotrichaceae decreased after POCD and increased after OI treatment, while Lachnospiraceae increased after POCD and decreased after OI treatment. **c** β-diversity by PCoA. **d** Chao1 index to characterize richness, Simpson and Shannon indices to characterize diversity, and OI treatment after POCD decreased richness and diversity (*p < 0.05 vs. POCD, one-way ANOVA). **e**, **f** Gut microbial markers identified by LEfSe analysis. **g**, **h** PICRUSt analysis determined the potential function of the gut microbiota in the administration of OI therapy after POCD. n = 5 for 16S rRNA gene sequencing

We performed 16S rRNA gene sequencing to assess the effects of OI treatment on gut microbiota dysbiosis in aged mice with POCD 24 h after surgery. Multilevel analysis of differences in gut flora composition showed that at the phylum level (Fig. 3a), the p_ Bacilli and p_Bacteroidia phyla had the highest abundances, followed by p_Clostridia, in the three groups of mice. The number of p_Bacilli decreased in the POCD group and increased in the OI group. Compared with the POCD group, p_Bacteroidia and p_Clostridia decreased in the OI group. At the genus level (Fig. 3b), g_Lactobacillaceae and g_Erysipelotrichaceae were the most abundant genera in the three groups. g_Lactobacillaceae and g_Erysipelotrichaceae were significantly less abundant in the POCD group than in the OI group, whereas g_Muribaculaceae was significantly more abundant in the OI group. The results showed that, in order of relative abundance, p_Bacteroidia, p_Clostridia, g_Lactobacillaceae, g_Erysipelotrichaceae, and g_Muribaculaceae were the dominant intestinal flora in the three groups of aged mice.

The results of the α-diversity analysis showed that the OI group had altered richness and diversity of the intestinal flora. We analyzed the changes in β-diversity using principal component analysis (PCoA) (Fig. 3c). Significant differences were found between the POCD and OI groups, except for a crossover between the Sham and POCD groups. To thoroughly evaluate the α-diversity of microbial communities, we utilized the Chao1 index to assess richness and the Simpson and Shannon indices to evaluate diversity (Fig. 3d). The results showed that OI significantly altered the diversity and composition of the gut microbiota. These data suggest that OI can alter the diversity and composition of the gut flora in POCD mice.

To further investigate the changes in gut microbiota biomarkers after drug administration, we performed linear discriminant analysis of effect size (LEfSe) to identify microbial markers (Fig. 3e, f). Compared with the Sham group, the POCD group was mainly enriched in o_Bacteroidales, c_Bacteroidia, and p_Bacteroidota; compared with the POCD group, the OI group was mainly enriched in o_Erysipelotrichaceae, g_LLeibacterium, and f_Erysipelotrichaceae. We used PICRUSt to analyze the potential functions of the gut microbiota (Fig. 3g, h). Compared with the POCD group, OI intervention altered amino acid metabolism, carbohydrate metabolism, metabolism of cofactors and vitamins, metabolism of terpenoids and polyketides, nucleosides, and nucleotide biosynthesis. OI treatment led to functional differences in the gut microbiota of aged POCD mice.

### Altered metabolomics of OI after the development of POCD in aged mice

**Fig. 4:**
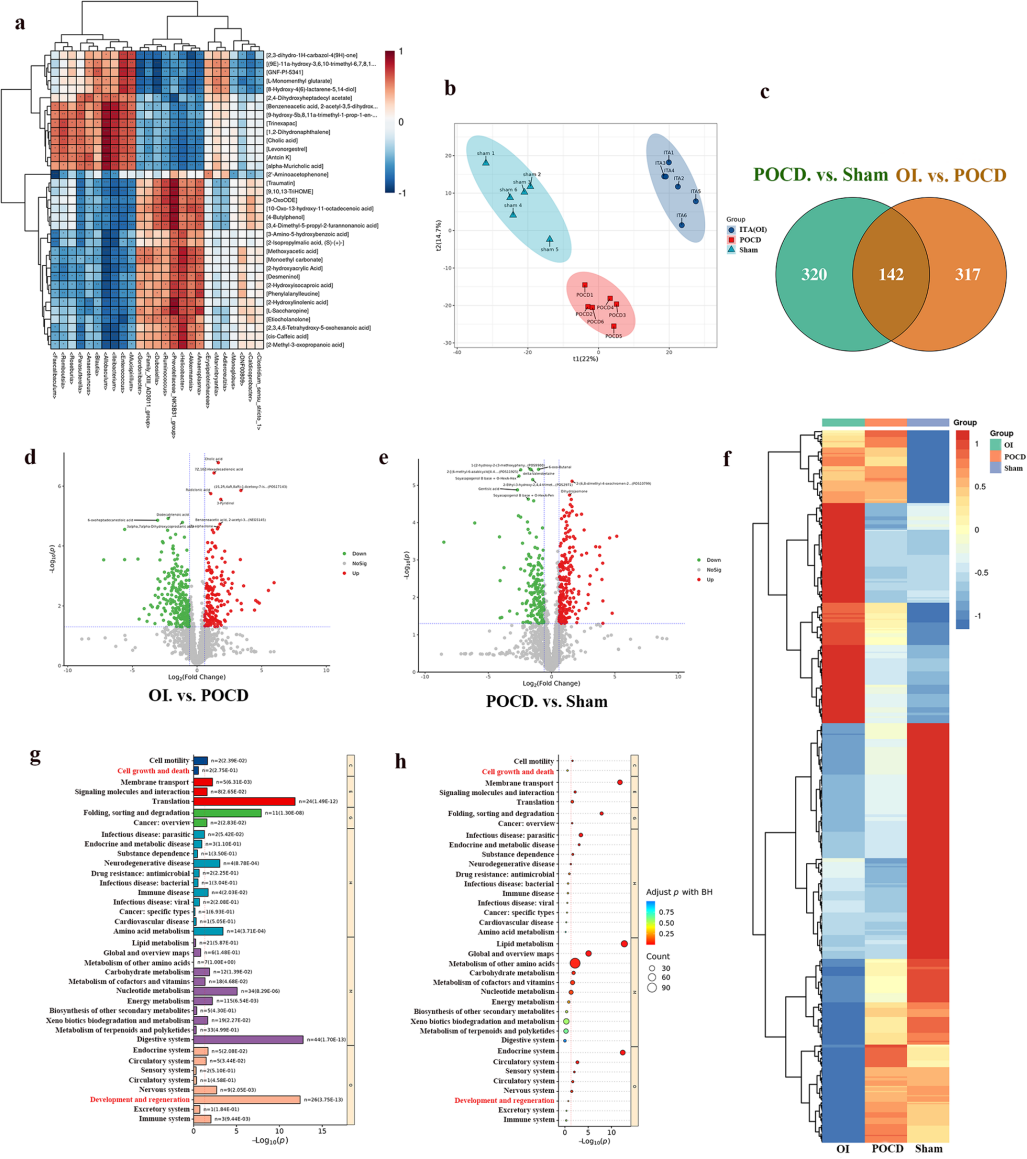
OI treatment improves POCD in aged mice by modulating neurogenesis. The intestinal contents of aged mice were subjected to an untargeted metabolomic analysis. **a** Correlation hierarchical clustering heatmap showing the direct correlation between gut flora and metabolites in aged mice among the three groups. **b** Partial least squares discriminant analysis score plot showing good separation between the sham, POCD, and OI groups. **c**–**f** Differentially expressed metabolites were identified by Venn diagram (**c**), volcano plots (**d**, **e**) and heatmap (**f**). **g**, **h** Kyoto Encyclopedia of Genes and Genomes (KEGG) analysis evaluated the enriched pathways for the representative profiles of these differential metabolites. *n* = 5 for an untargeted metabolomic analysis

Considering the interplay between the intestinal flora and host metabolism, an untargeted metabolomic analysis was subsequently performed on samples subjected to 16S rRNA gene sequencing to investigate the metabolomic profiles after OI treatment. The two histological elements were subjected to Spearman’s correlation hierarchical clustering analysis by conjoint analysis, which showed that changes in the number of flora after OI treatment led to changes in the number of corresponding metabolites (Fig. 4a). The partial least squares discriminant analysis score plot demonstrated clear separation between the Sham, POCD, and OI groups, indicating successful modeling of POCD and showing that the OI treatment resulted in alterations in intestinal metabolites (Fig. 4b). A total of 462 metabolites were identified in the POCD and sham groups, whereas 459 metabolites were identified in the OI and POCD groups. A total of 142 metabolites were identified in the three groups (Fig. 4c–f). The differential metabolites showed significant functional enrichment in cell growth, death, development, and regeneration based on Kyoto Encyclopedia of Genes and Genomes analysis (Fig. 4g, h), indicating that the regulation of neurogenesis may be involved in OI-mediated neuroprotection against POCD.

### OI enhances POCD recovery by promoting endogenous neurogenesis.

**Fig. 5:**
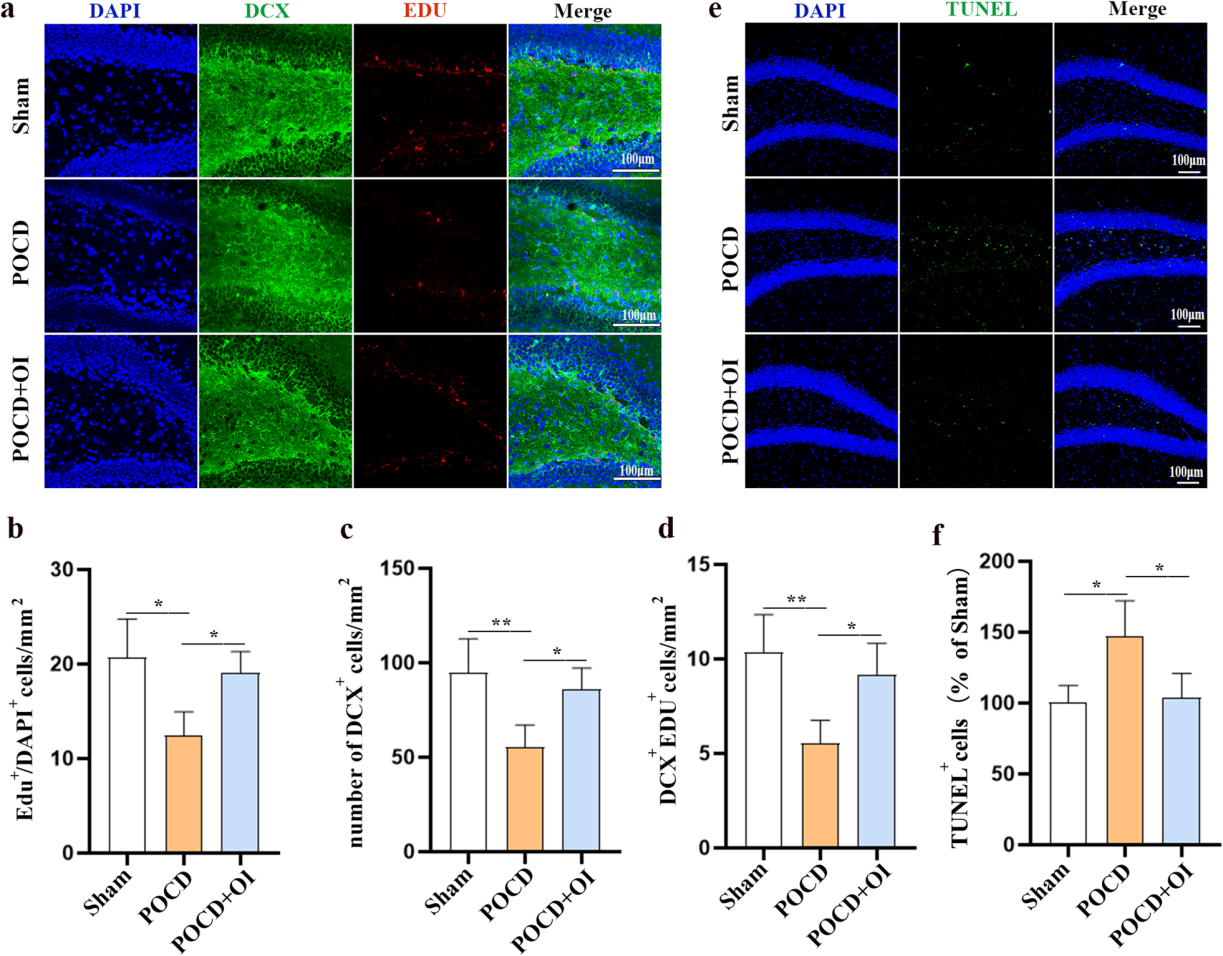
OI treatment promotes neurogenesis and reduces neuronal apoptosis. **a** Representative immunostaining images of DCX (green) and EDU (red) in the hippocampal DG region. **b**–**d** Quantification of DCX and EDU positively stained cells. **e** Representative TUNEL immunostaining images in the hippocampal DG region. **f** Statistics of TUNEL-positive cells. Data are expressed as mean ± SD. *n* = 4. *P < 0.05, **P < 0.01, and ***P < 0.001 by one-way ANOVA

To explore the potential of OI in promoting endogenous neurogenesis, we employed DCX and EDU to mark newly generated neurons and observed neurogenesis in the dentate gyrus (DG) region of the hippocampus in a separate cohort of aged mice on postoperative day 9. Immunofluorescence staining results showed that the number of newborn neurons in the hippocampal DG area of aged mice in the POCD model group was significantly reduced compared to that in the Sham group (EDU^+^, 12.40 ± 2.533 vs 19.03 ± 2.284 cells/mm^2^, *P* = 0.0108, DCX^+^, 55.15 ± 11.87 vs 94.52 ± 18.18 cells/mm^2^, *P* = 0.0085, EDU^+^DCX^+^, 5.518 ± 1.237 vs 10.33 ± 2.033 cells/mm^2^, *P* = 0.0074) and the number of newborn neurons in the hippocampal DG area of aged mice in the OI treatment group was significantly higher than that in the POCD group (EDU^+^, 12.40 ± 2.533 vs 19.03 ± 2.284 cells/mm^2^, *P* = 0.0337, DCX^+^, 55.15 ± 11.87 vs 86.03 ± 11.17 cells/mm^2^, *P* = 0.0310, EDU^+^DCX^+^, 5.518 ± 1.237 vs 9.149 ± 1.687 cells/mm^2^, *P* = 0.0335) (Fig. 5a–d). To verify the effect of OI treatment on neuronal survival, TUNEL staining was utilized. Neuronal apoptosis was elevated in aged mice in the POCD model group in comparison to the Sham group (146.8 ± 25.37 vs 100.0 ± 12.25%, *P* = 0.0170). However, after the administration of OI, neuronal apoptosis was significantly reduced (103.9 ± 16.99 vs146.8 ± 25.37%, *P* = 0.0265), indicating a protective effect on neuronal survival (Fig. 5e, f). Our results showed that OI treatment induces endogenous neurogenesis and attenuates neuronal apoptosis.

### Nrf2/ERK signaling is involved in OI-mediated endogenous neurogenesis in POCD

**Fig. 6:**
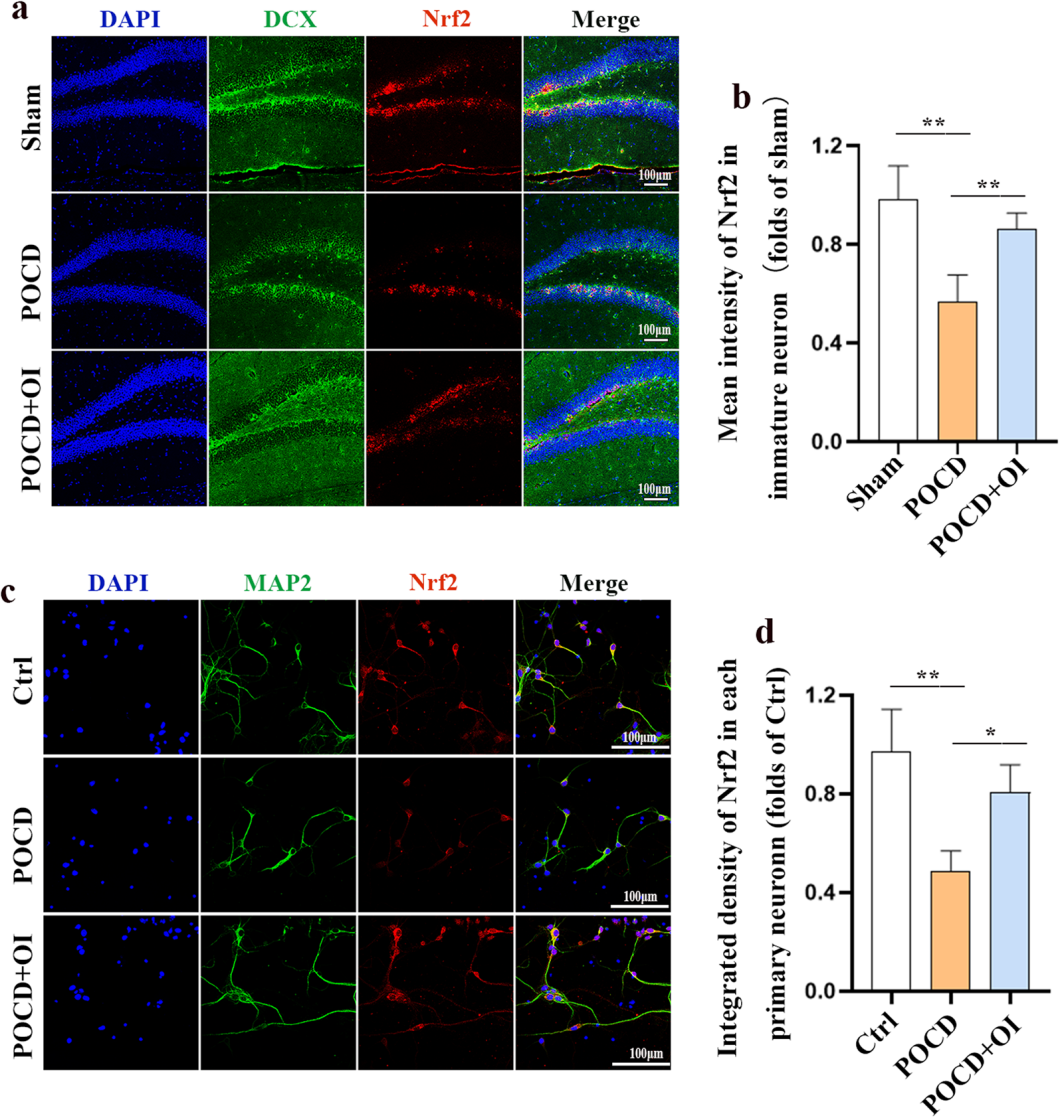
Nrf2 expression is increased in hippocampal neurons after OI treatment. **a** Representative immunofluorescence staining of DCX (green) and Nrf2 (red) in the hippocampal DG region. **b** Quantification of Nrf2 intensity in immature neurons in the DG region. **c** Immunofluorescence staining of Nrf2 (red) and MAP2 (green) in primary neurons. **d** Quantification of integrated density of Nrf2 in the primary neurons. Data are expressed as mean ± SD (*n* = 4), *P < 0.05, **P < 0.01, and ***P < 0.001 by one-way ANOVA

**Fig. 7:**
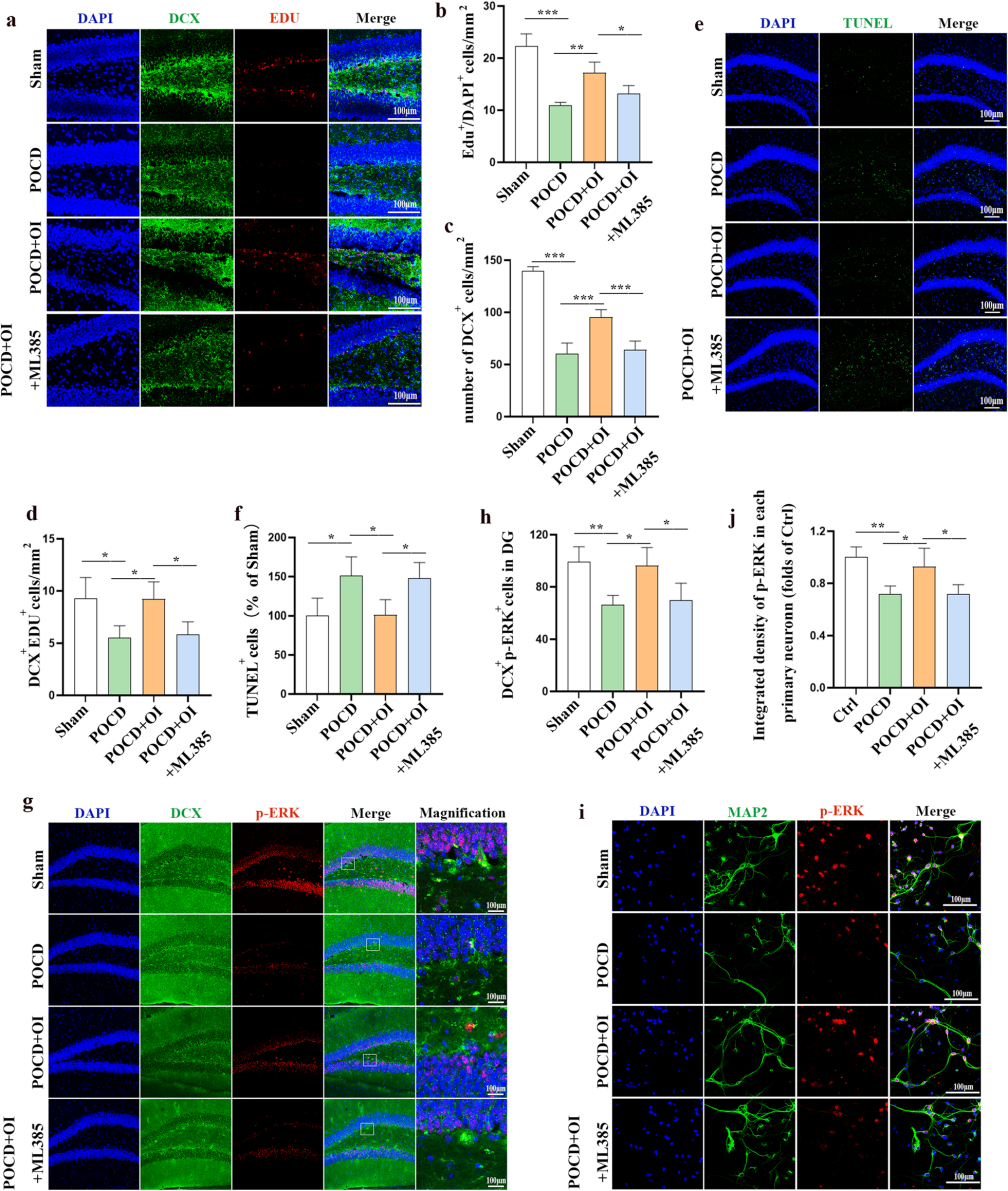
The Nrf2/ERK signaling pathway is involved in mediating OI-driven neurogenesis. **a** Representative immunostaining images of DCX (green) and EDU (red) in the hippocampal DG region. **b**–**d** Quantification of EDU and DCX positively stained cells. **e** Representative TUNEL immunostaining images in the hippocampal DG region. **f** Statistics of TUNEL-positive cells. **g** Representative immunostaining images of DCX (green) and p-ERK (red) in the hippocampal DG region. **h** Quantification of DCX and p-ERK positively stained cells. **i** Immunofluorescence staining of p-ERK (red) and MAP2 (green) in primary neurons. **j** Quantification of integrated density of p-ERK in the primary neurons. Data are expressed as mean ± SD. *n* = 4. *P < 0.05, **P < 0.01, and ***P < 0.001 by one-way ANOVA

Considering that the activation of Nrf2 signaling by OI can attenuate inflammation and oxidative stress, we hypothesized that OI may improve POCD to a certain extent by activating Nrf2 related signaling. To verify this, we first tested Nrf2 expression levels in hippocampal neurons in the POCD model through both in vivo and in vitro experiments and found that Nrf2 expression was downregulated in hippocampal neurons 9 days after anesthesia and surgery (0.5696 ± 0.1073 vs 0.9822 ± 0.1362, *P* = 0.0011), and Nrf2 expression was increased in hippocampal neurons after OI treatment (0.8618 ± 0.06596 vs 0.5696 ± 0.1073, *P* = 0.0097) (Fig. 6a, b). Similarly, LPS and isoflurane reduced Nrf2 expression in primary neurons 24 h after stimulation (0.4850 ± 0.08347 vs 0.9709 ± 0.1723, *P* = 0.0012), and this reduction was reversed by OI treatment (0.8078 ± 0.1106 vs 0.4850 ± 0.08347, *P* = 0.0149) (Fig. 6c, d). To further determine whether Nrf2 signaling mediates the promotion of neurogenesis by OI treatment of POCD in aged mice, mice were intraperitoneally administered the Nrf2 antagonist ML385 in the following experiment, and the same amount of normal saline was administered intraperitoneally to mice in the other groups. The results showed that ML385 significantly reversed the increase in the number of newborn neurons (EDU^+^, 13.19 ± 1.515 vs 17.15 ± 2.073 cells/mm^2^, *P* = 0.0350, DCX^+^, 63.83 ± 8.636 vs 95.40 ± 7.204 cells/mm^2^, *P* = 0.0005, EDU^+^DCX^+^, 5.819 ± 1.243 vs 9.215 ± 1.651 cells/mm^2^, *P* = 0.0389) (Fig. 7a–d) and the decrease in the number of neuronal apoptosis (148.2 ± 19.92 vs 101.3 ± 19.13%, *P* = 0.0397) (Fig. 7e, f) in the hippocampal DG area of aged mice in the POCD + OI + ML385 group compared with the POCD + OI group. Given that the activation of ERK enhances neurogenesis and that Nrf2 serves as a potential regulator of ERK [28, 29], we speculated that the activation of the Nrf2/ERK signaling pathway may underlie the pro-neurogenic effects of OI on POCD. We assessed ERK phosphorylation in immature neurons using phospho (p)-ERK^+^ and DCX^+^ immunostaining 9 days after surgery following the administration of OI and ML385. The results revealed that anesthesia and surgery led to a significant decrease in the number of DCX^+^ p-ERK^+^ cells in the DG region of the hippocampus (66.18 ± 7.150 vs 99.49 ± 11.29 cells/mm^2^, *P* = 0.0074), which was reversed by OI treatment (66.18 ± 7.150 vs 96.22 ± 13.93 cells/mm2, *P* = 0.0148). However, the administration of ML385 reversed the OI-induced increase in p-ERK in immature neurons in the DG (69.89 ± 12.82 vs 96.22 ± 13.93 cells/mm2, *P* = 0.0327) (Fig. 7g, h). In in vitro experiments, LPS and isoflurane reduced p-ERK expression in primary neurons 24 h after stimulation (0.7153 ± 0.06457 vs 1.000 ± 0.07916, *P* = 0.0050), and this reduction was reversed by OI treatment (0.7153 ± 0.06457 vs 0.9280 ± 0.1411, *P* = 0.0333). Blocking Nrf2 activation with ML385, however, exacerbated the decrease in p-ERK expression in primary neurons treated with OI (0.7183 ± 0.07025 vs 0.9280 ± 0.1411, *P* = 0.0360) (Fig. 7i, j). These findings suggested that OI treatment may promote endogenous neurogenesis through the Nrf2/ERK signaling pathway.

### ML385 reverses the neuroprotective effects of OI on POCD

**Fig. 8:**
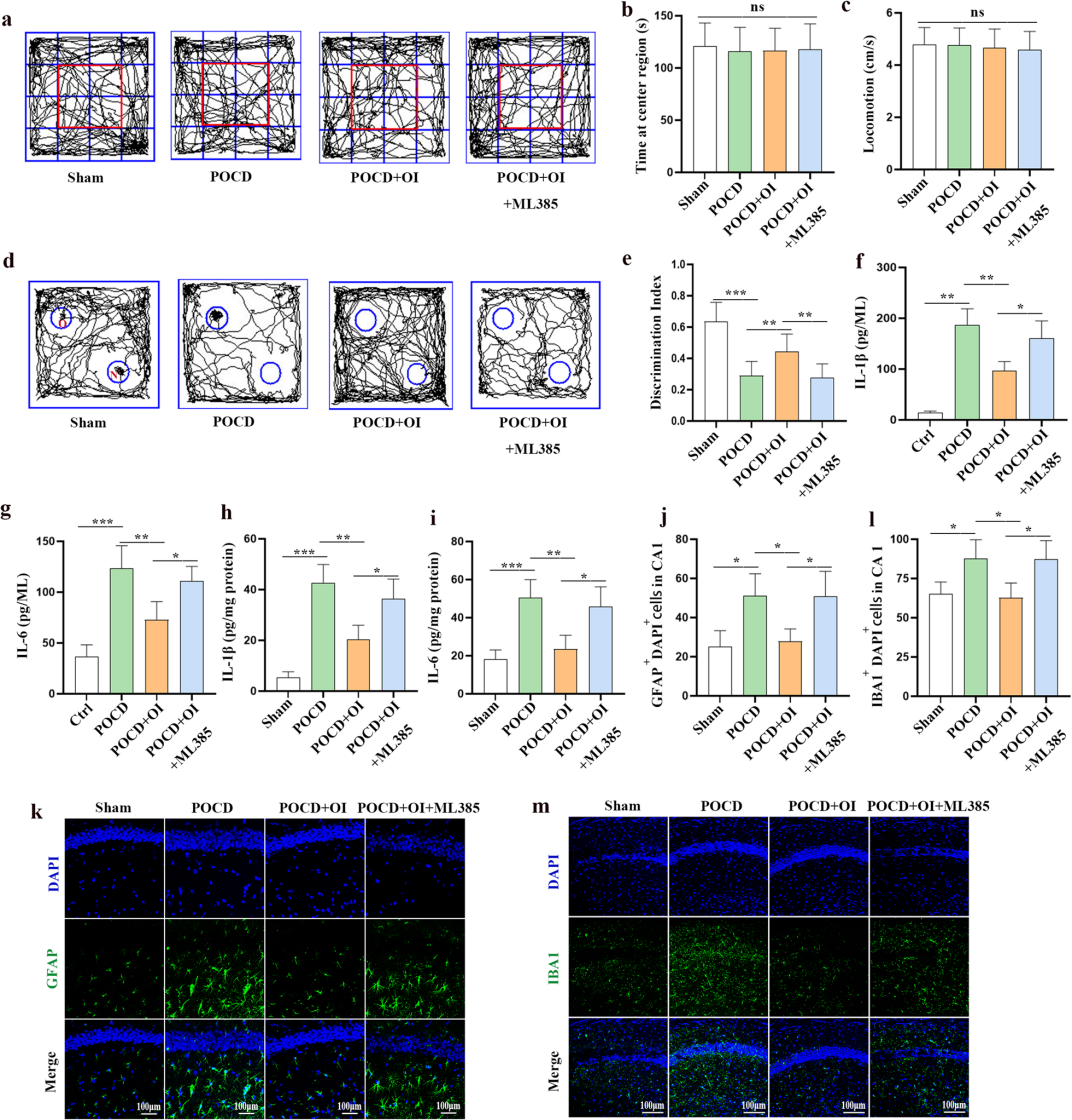
Inhibition of Nrf2 signaling by ML385 weakens the capacity of OI to reverse POCD and inhibit neuroinflammation in the hippocampus. **a**–**c** Open field test (OFT) was used to detect anxiety and motor activity in aged mice; **a** Road map of the OFT, **b** Time spent in the center region, **c** average velocity in the OFT. **d**, **e** The NOR test was performed to measure recognition memory; **d** The typical routes in the NOR tests, **e** Discrimination index. Determination of IL-1β (**f**) and IL-6 (**g**) levels by ELISA in primary microglia. Determination of IL-1β (**h**) and IL-6 (**i**) levels by ELISA in the hippocampus of aged mice. Representative immunofluorescence staining and Statistics of GFAP (**j**, **k**) and Iba1 (**l**, **m**) in the hippocampal CA1 region of aged mice. Data are expressed as mean ± SD, *n* = 12 for behavioral experiments, *n* = 4 for inflammatory cytokines analysis. *P < 0.05, **P < 0.01, and ***P < 0.001 by one-way ANOVA. ns, no significant difference

To further determine whether Nrf2 signaling mediates the neuroprotective effects of OI treatment on POCD in aged mice, mice were intraperitoneally administered ML385 in the following experiment. The OFT and NOR tests were performed 5–6 days after surgery. In the OFT, no significant difference was observed in the time spent in the center region (F _(3, 44)_ = 0.1033, *P* = 0.9577) and locomotion (F _(3, 44)_ = 0.2175, *P* = 0.8837) among the four groups, suggesting that exploratory laparotomy under isoflurane anesthesia did not significantly cause anxiety behaviors or damage motor activity in aged mice (Fig. 8a–c). In the NOR test, OI-treated mice spent more time exploring new objects than mice in the POCD group at 1 h after the initial training sessions. However, intraperitoneal injection of ML385 abolished the therapeutic effects of OI (0.2750 ± 0.08960 vs 0.4425 ± 0.1131, *P* = 0.0018) (Fig. 8d, e). To demonstrate whether ML385 reversed the role of OI in reducing neuroinflammation, we measured the expression of IL-1β and IL-6 in hippocampal neurons (Fig. 8f, g) and brain tissues (Fig. 8h, i) in both the in vivo and in vitro models. The results showed that ML385 reversed the inhibitory effects of OI on L-1β (160.2 ± 34.51 vs 96.34 ± 18.50 pg/ml, *P* = 0.0168) and IL-6 (110.9 ± 14.42 pg/ml vs 72.79 ± 17.95 pg/ml, *P* = 0.0355) expression in primary microglia subjected to LPS and isoflurane treatment. Similarly, compared with mice in the POCD + OI group, the expression of IL-1β (36.37 ± 7.809 vs 20.26 ± 5.713 pg/mg, *P* = 0.0133) and IL-6 (45.82 ± 10.38 vs 23.48 ± 7.269 pg/mg, *P* = 0.0111) were increased in mice of POCD + OI + ML385 group 24 h after surgery, and immunofluorescence staining results showed a significant increase in microglia and astrocytes labeled by Iba1 (87.41 ± 11.70 vs 62.61 ± 9.535 cells, *P* = 0.0246) and GFAP (50.80 ± 12.80 vs 27.93 ± 6.269 cells, *P* = 0.0312), respectively, in the POCD + OI + ML385 group compared to the POCD + OI group. Therefore, OI treatment may improve POCD by activating Nrf2 signaling (Fig. 8j–k).

## Discussion

**Fig. 9:**
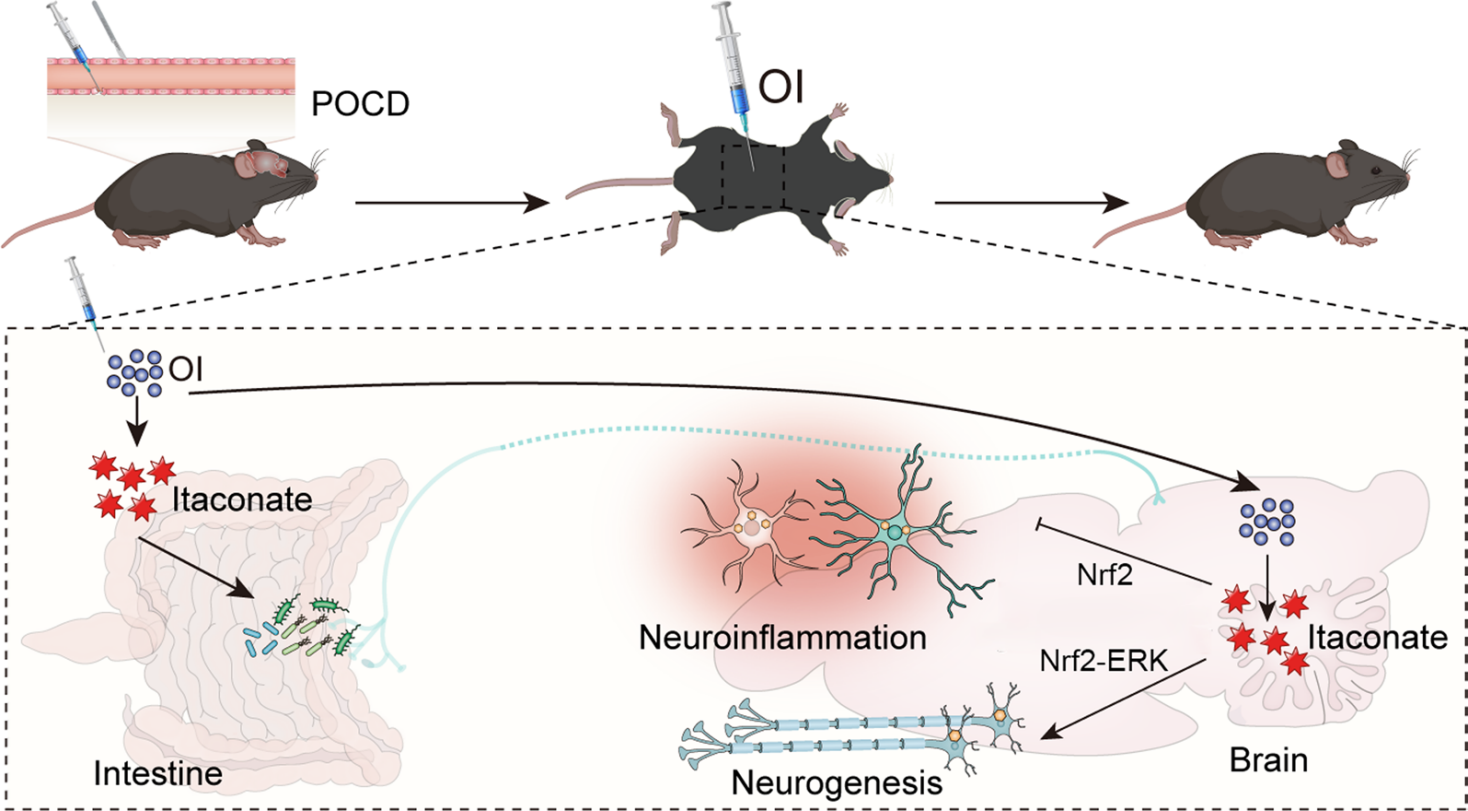
Working hypothesis for OI-mediated therapeutic effect on POCD in aged mice. OI can be hydrolyzed to itaconate after administration, which may reverse anesthesia/surgery-induced gut dysbiosis to produce neuroprotective metabolites. OI may enter the brain parenchyma due to its high cell permeability and activate Nrf2 signaling to inhibit neuroinflammatory responses and restore neurogenesis by hydrolyzing into itaconate. These combined mechanisms contributed to cognitive improvement in aged mice with POCD. OI, 4-octyl itaconate; POCD, postoperative cognitive dysfunction; Nrf2, nuclear factor erythroid 2-related factor 2; ERK, extracellular signal-related kinases

In this study, we investigated the therapeutic effects of itaconate derivative on POCD and explored the potential mechanisms. Our findings suggest that OI can ameliorate anesthesia/surgery-induced cognitive impairment, as demonstrated by the improved performance of aged mice in the OI treatment group compared to the POCD group in the NOR test. Moreover, the mechanism behind this improvement may be associated with OI's ability to reverse anesthesia/surgery-induced intestinal dysbiosis and activate Nrf2 signaling-dependent anti-neuroinflammatory and pro-neurogenic responses (Fig. 9). This study indicates that OI holds promise as a therapeutic agent for the treatment of POCD.

Currently, the clinical prevention and treatment of POCD mainly focus on controlling its high-risk factors and optimizing organ function during the perioperative period [30]. Risk factors for POCD include susceptibility and triggering factors [31]. Susceptibility factors are baseline factors include advanced age (> 65 years), frailty, lower level of education, preoperative cognitive decline or dementia, carrying the apolipoprotein E ε4 variant, and metabolic syndrome (obesity, diabetes, etc.) [32]. Triggering factors occur throughout the perioperative period and include the use of preoperative anticholinergic drugs, emergency surgery, prolonged surgery duration, surgery for major trauma, postoperative admission to the intensive care unit (ICU), postoperative infection, and cardiovascular events [31]. Individualized comprehensive prevention and treatment based on controllable risk factors are the main strategies for preventing and treating POCD in clinical practice [30, 32]. These strategies include adjusting the patient’s medications before surgery, optimizing the organ function status of elderly patients, and improving metabolic syndrome [33]. It also involves selecting the most appropriate anesthesia and surgical approach and controlling the depth of anesthesia guided by the bispectral index during general anesthesia to avoid burst suppression, maintain hemodynamic stability, and avoid significant fluctuations in circulation [30]. Postoperatively, controlling patient pain and minimizing ICU admissions and infections are important [31]. However, despite the benefits of these comprehensive prevention and treatment strategies, there is a lack of direct evidence demonstrating their impact on the incidence of POCD. Furthermore, in the specific implementation process, the clinical effectiveness of this strategy is closely related to the individual experience and clinical skills of anesthesiologists, making it difficult to generalize the results to clinical practice. Therefore, recent clinical and basic studies have focused on identifying simple and effective ways to prevent and treat POCD.

Inflammatory mechanisms are currently recognized as the pathogenic mechanisms underlying POCD. Chronic low-grade inflammation in the aging brain increases the susceptibility of the brain to external insults [34]. Surgical trauma and anesthetics can activate peripheral immune cells (such as macrophages) and trigger systemic inflammation. Peripheral inflammation and immune cells can penetrate the damaged blood–brain barrier (BBB) and enter the brain, activating microglial cells and inducing cognitive impairment [2, 35, 36]. Microglia are a key cell type influenced by anesthesia and surgery and play a central role in regulating neuroinflammation. Once activated, microglia exhibit significant morphological changes, release a large amount of pro-inflammatory factors, and can activate astrocytes to amplify neuroinflammation [35, 37]. Abundant clinical and basic research has confirmed the critical roles of inflammatory mediators IL-1β and IL-6 in the occurrence and development of POCD. Surgical trauma can induce a sterile inflammatory response in the peripheral immune system, leading to the massive release of inflammatory mediators into the blood, subsequently inducing central nervous system overexpression of IL-1β, triggering cognitive impairment [38]. Most recent studies and drug development efforts for POCD treatment are focused on the pro-inflammatory effects of anesthesia and surgery [2, 35]. A series of specific and potent anti-inflammatory therapeutic drugs and methods have been identified and developed, including specific pro-resolving inflammatory mediators (such as Maresin 1 [39]) and cholinergic agonists (such as nicotine [40]). Despite extensive basic research exploring various small molecule neuroprotective agents and approaches targeting inflammation for the prevention and treatment of POCD over the past few decades, the clinical translation progress has been slow [35, 41].

Itaconate and its derivatives, including OI and dimethyl itaconate, are emerging as regulators of the inflammatory cascade; however, their potential and precise mechanisms in POCD remain poorly understood. Itaconate is the most abundant metabolite in LPS-induced macrophages [9]. Recent studies have demonstrated the role of itaconate in suppressing bacterial infections and pathogen replication. During microbial invasion, itaconate effectively restricts the growth of *Pseudomonas indigofera* by inhibiting isocitrate lyase activity, and its metabolite, itaconyl-CoA, also inhibits pathogen growth after *Mycobacterium tuberculosis* infection by regulating methylmalonyl-CoA mutase [42, 43]. Moreover, itaconate potently restores the integrity of the intestinal barrier and alleviates dysbiosis of the gut microbiota in a model of hypervirulent *Klebsiella pneumoniae* infection [44]. Supplementation with dimethyl itaconate improves microbiome alterations and attenuates high-fat diet-mediated cognitive decline in mice [45]. Previous evidence has determined an association between gut microbiota alterations and POCD development, and stabilizing the gut microbiota could reduce neuroinflammation and attenuate POCD [25]. Our experiments revealed that OI administration mitigated anesthesia/surgery-induced disturbances in intestinal dysbiosis of the gut microbiota, which may contribute to the therapeutic effects of itaconate. Itaconate has emerged as a crucial component in host anti-inflammatory effects, and exogenous supplementation with OI has also shown immunomodulatory effects on macrophage activation and inflammation through diverse mechanisms, including protein modification of cysteine residues and suppression of enzymatic activity [9, 46, 47]. Due to its significant polarity, it is improbable for itaconate to penetrate the BBB [9]. OI exhibits reduced polarity which enhances cell permeability, thereby increasing the likelihood of crossing the BBB [16]. Treatment with OI or other derivatives has been shown to improve many neurological disorders, including ischemic intracerebral hemorrhagic stroke [46], high-fat diet-induced cognitive impairment [45], and Alzheimer’s disease [48, 49] via anti-inflammatory effects. Here, we demonstrated that OI administration significantly attenuated the cognitive impairment induced by anesthesia/surgery. Moreover, we found that OI treatment could inhibit glial activation and reduced the expression of the two key inflammatory cytokines IL-1β and IL-6 in the hippocampus after anesthesia and surgery. These data provide evidence that OI treatment may improve POCD by regulating alterations in gut microbiota and inhibiting neuroinflammation.

Adult neurogenesis is associated with the maintenance of neural plasticity and cognitive recovery [50]. Neural stem cells form the basis of adult neurogenesis and can differentiate into immature neurons under appropriate stimuli, which can mature further into fully functional neurons [51]. DCX is a specific marker of immature neurons, and its expression level reflects the number of immature neurons in the brain. In a nonpathological state, these immature neurons can develop into mature neurons under changes in the surrounding environment or external interventions [52]. Recent studies have shown that surgery can significantly reduce the number of immature neurons in the mouse brain [53, 54]. The results of untargeted metabolomics of the intestinal flora show that OI treatment causes differential metabolites associated with cell growth, death, development, and regeneration. Therefore, we investigated whether OI administration affects apoptosis and endogenous neurogenesis in mice after anesthesia and surgery by analyzing DCX expression in newly generated immature hippocampal neurons. This study suggests that anesthesia and surgery cause neuronal apoptosis and downregulate the number of newly generated DCX^+^ immature neurons in the hippocampal DG area, whereas OI supplementation significantly inhibits neuronal apoptosis and increases the number of new immature neurons, indicating that OI may improve POCD by promoting neurogenesis by regulating the gut microbiota. Nrf2, a transcription factor of the Cap’n’collar subfamily, is required for itaconate to exert anti-inflammatory effects. Endogenous itaconate is synthesized and then migrates to the cytosol to activate Nrf2 and initiate transcriptional antioxidant and anti-inflammatory actions [9]. Nrf2 is normally involved in neurogenic processes and promotes the regulation of neural stem cells to neuronal fate commitment and differentiation [55]. OI has been reported to produce functional itaconate by hydrolysis and boost Nrf2 protein levels in macrophages [9]. Our results show that Nrf2 was upregulated in neurons after OI administration. In support of the role of Nrf2 in the effects of OI, intraperitoneal injection of a Nrf2 antagonist reversed the neurogenesis of old mice with OI treatment. A Nrf2 antagonist also increased neuroinflammation and worsened cognitive function in mice treated with OI. Given that activation of Nrf2 can initiate various downstream pathways, we investigated the involvement of ERK, a pivotal molecule crucial for the regulation of neurogenesis [28], cognition [29] and POCD [56], in the neuroprotective effects of OI on POCD. Importantly, ERK has been identified as a regulator of Nrf2 [57–59]. In the present study, OI treatment induced the activation of the Nrf2/ERK signaling pathway, leading to increased phosphorylation of ERK in immature neurons. However, administration of ML385 reversed these changes. These findings underscore the crucial role of the Nrf2/ERK signaling pathway following OI treatment in POCD.

## Conclusions

Our study suggests a therapeutic role for the exogenous itaconate derivative, OI, in POCD. The mechanism may be associated with the inhibition of neuroinflammation and promotion of neurogenesis via the activation of Nrf2 signaling and the stabilization of the gut microbiota. The study provides a novel approach for developing potential interventions for POCD by either enhancing endogenous itaconate production or supplementing with exogenous itaconate derivatives.

## Data Availability

The datasets presented in this study can be found in online repositories. The names of the repository/repositories and accession number (s) can be found below: NCBI BioProject (https://www.ncbi.nlm.nih.gov/sra/PRJNA1078590) and MetaboLights repository (https://www.ebi.ac.uk/metabolights/editor/study/MTBLS9593). Other datasets used and analyzed during this study can be obtained from the corresponding author upon reasonable request.
